# Supplementary material for: The Construction and Exploration of a Comprehensive MicroRNA Centered Regulatory Network in Foxtail Millet (Setaria italica L.)
Source: Front Plant Sci. 2022 May 6;13:848474. doi: 10.3389/fpls.2022.848474 (PMC9121102; doi:10.3389/fpls.2022.848474)
Supplement: Supplementary file 6 [file Data_Sheet_6.zip › Sit-miR397_Seita.9G510100.1_2364_TPlot.pdf]

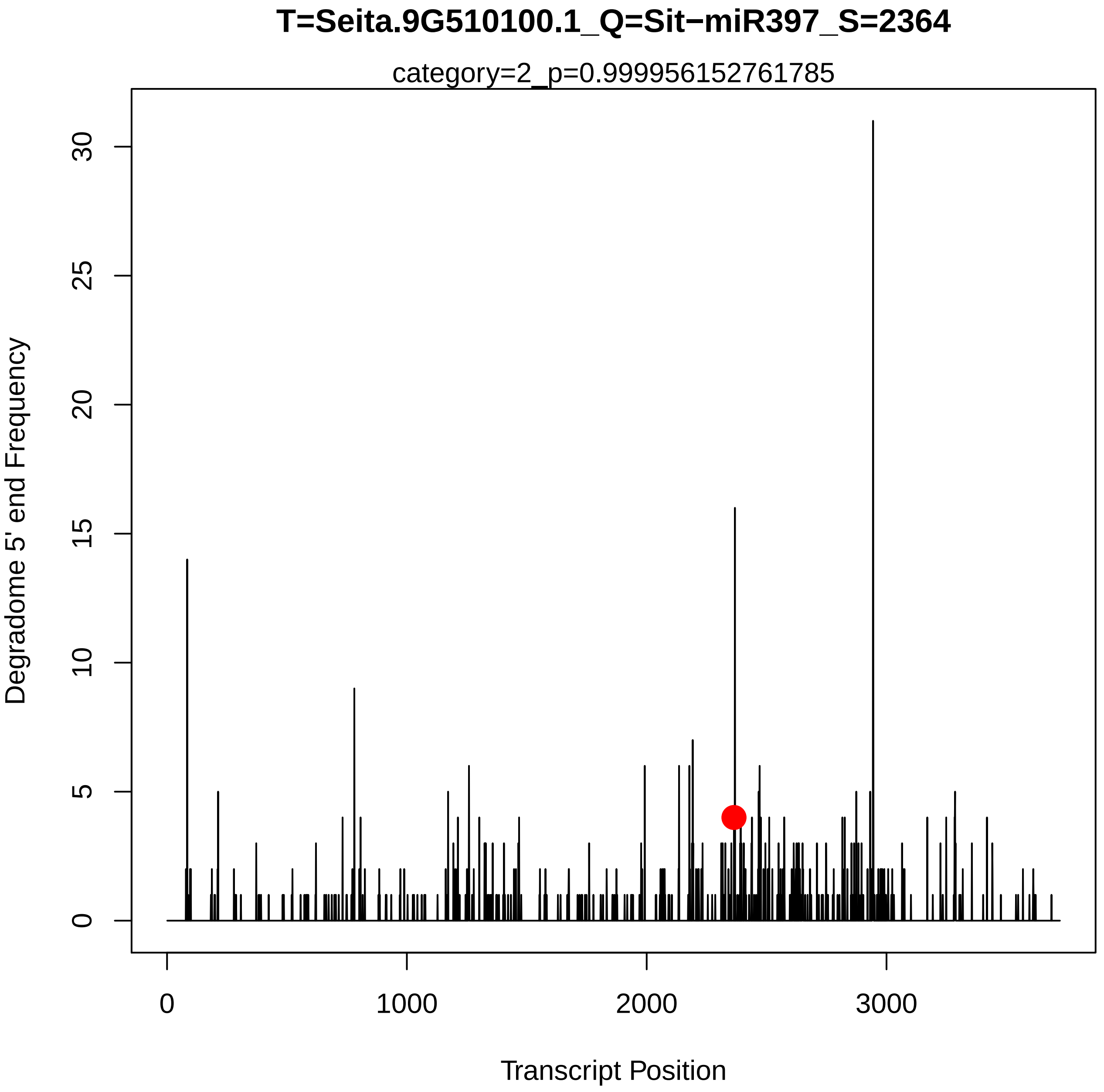
T=Seita.9G510100.1_Q=Sit−miR397_S=2364
category=2_p=0.999956152761785
Degradome 5' end Frequency
0
5
10
15
20
25
30
0
1000
2000
3000
Transcript Position
